# Is feedback to medical learners associated with characteristics of improved patient care?

**DOI:** 10.1007/s40037-017-0375-8

**Published:** 2017-08-29

**Authors:** Victoria Hayes, Robert Bing-You, Kalli Varaklis, Robert Trowbridge, Heather Kemp, Dina McKelvy

**Affiliations:** Maine Medical Center, Portland, ME USA

**Keywords:** Feedback, Medical Education, Patient care

## Abstract

**Purpose:**

To investigate the association of medical learner feedback with patient management and outcomes.

**Methods:**

The authors investigated 27 articles that utilized patient data or chart reviews as a subset of a prior feedback scoping review. Data extraction was completed by two authors and all authors reviewed the descriptive data analysis.

**Results:**

The studies were predominantly short-term investigations conducted in the US at academic teaching hospitals (89%) with one medical discipline (78%), most commonly internal medicine (56%). Patient-related outcomes primarily involved improved documentation (26%) and adherence to practice guidelines (19%) and were mostly measured through chart reviews (56%) or direct observation (15%). The primary method of feedback delivery involved a written format (30%). The majority of the studies showed a positive effect of feedback on the patient-oriented study outcomes (82%), although most involved a non-rigorous study design.

**Conclusions:**

Published studies focusing on the relationship between medical learner feedback and patient care are sparse. Most involve a single discipline at a single institution and are of a non-rigorous design. Measurements of improved patient outcomes are restricted to changes in management, procedures and documentation. Well-designed studies that directly link learner feedback to patient outcomes may help to support the use of feedback in teaching clinical outcomes improvement in alignment with competency-based milestones.

**Electronic supplementary material:**

The online version of this article (10.1007/s40037-017-0375-8) contains supplementary material, which is available to authorized users.

## What this paper adds

Feedback is a central topic in medical education that has been deemed essential for helping learners to improve performance and meet standards of competency. In an educational system that strives to focus on both academic milestones and clinical outcomes, patient care has become a prominent feature. It is unclear, however, what is known about feedback to medical learners and its association with patient-related outcomes. Our analysis describes the state of the medical education literature on the topic of feedback to medical learners and its association with characteristics of improved patient care and provides a description of areas in need of further investigation.

## Introduction

Feedback has been a focus of the medical education literature for decades, covering a variety of features, techniques and purposes. Feedback has been deemed essential to promoting learning [1], improving performance [2], acquiring clinical skills [3] and more recently, meeting standards of competency [4]. Feedback is an important component of formative assessment (the exchange of information designed to support development) [5]. The outcome of a summative assessment that measures success or proficiency can also be used as feedback to guide subsequent efforts [6]. Despite the importance of feedback and the attention it has received in scholarly literature, effective feedback remains difficult to achieve within the context of clinical education [5].

Many definitions of feedback exist in the literature, most focusing on behavioural change and performance improvement. A systematic review of feedback definitions in the medical literature by Van der Ridder [7] yielded the following compilation: “Feedback in clinical education is specific information about the comparison between a trainee’s observed performance and a standard, given with the intent to improve the trainee’s performance”. Is the focus of feedback as a means to effecting behavioural change enough? As recognized by Ende in his 1983 sentinel article on the use of feedback in medical education, “The goal of clinical training is expertise in the care of patients” [8]. Should there be a more robust feedback end goal of improved patient care and outcomes?

Improving the patient experience of care, as well as improving the health of populations and reducing healthcare costs, are core dimensions of the Triple Aim as outlined in 2008 by the Institute for Healthcare Improvement [9]. In 2012, the Accreditation Council for Graduate Medical Education responded with the Next Accreditation System [10]. This shift toward competency-based medical training through the use of educational milestones was designed as a way to focus the system on both educational and clinical outcomes. The topic of patient care features prominently in the milestone sub-competencies specific to each residency specialty [11] and includes well-defined patient outcomes. Assessing these outcomes will become increasingly important as healthcare moves toward a value-based system [12]. Effective feedback strategies are believed to be crucial to help medical learners achieve these outcomes [3]. Programs that provide clinical performance feedback to promote compliance with patient care-related quality measures are now being utilized at the practising physician level [13]. It is unclear, however, what is known about feedback and improved patient outcomes at the medical learner level. To that end, we sought to answer the question “What is the association of medical learner feedback with characteristics of improved patient care?”

A 2016 scoping review conducted by the authors [14] provided a broad map of the feedback literature for undergraduate and graduate medical learners. Scoping reviews are utilized to examine the extent of research activity in a topic area, determine the value of undertaking a full systematic review and summarize research findings, as well as identify gaps for further research focus as is the purpose of this study [15]. For the current analysis, a subset of the articles was analyzed utilizing an evidence synthesis mapping process [16, 17] to explore the relationship of medical learner feedback to patient management and outcomes.

## Methods

The data sources for the initial scoping review have been described in full previously [14]. In brief, six databases were searched (Ovid MEDLINE, CINAHL, ERIC, ProQuest Dissertations and Theses Global, Scopus, and Web of Science) as well as seven well-known medical education journals and the reference lists of 11 key articles on feedback. The search terms *feedback*; *feedback; psychological*; *medical students*; *assessment*; *self-assessment*; *internship and residency*; *resident*; *fellows*; *medical education*; *faculty*; *faculty; medical*; and *reflection *were utilized. The search was limited to articles published from 1 January 1980 to 31 December 2015. A data extraction form was completed that included components such as methodology, intervention type, focus of feedback content and outcome measures. Twenty-seven articles, a small segment of the total 650, were identified from that review by two of the authors (V. H., R. B.) who utilized patient data or chart reviews as outcomes measures to assess the clinical impact of the feedback interventions.

The authors developed a data extraction form based on review models found in the medical literature [17, 18]. Two of us (V. H., R. B.) reviewed all the studies independently and conducted the data extraction component. A third author (K. V.) reviewed and resolved any discrepancies noted.

A follow-up search was conducted on 27 March 2017 to assess for new relevant articles. The prior search terms were utilized with the addition of *chart reviews *and *patient outcomes *for the years 2016–2018 (to enable capture of articles published ahead of print). This search yielded 119 articles which were then assessed for inclusion by two of the authors (V. H., R. B.). No additional articles were found that utilized patient data to assess the impact of feedback interventions.

## Results

A total of 27 articles focused on feedback in medical learners and patient-related outcomes, with years of publication ranging from 1980 to 2015, were reviewed in-depth (see Table 1 of the online Electronic Supplementary Material (ESM)) [19–45].

### Patient-related outcomes

The vast majority of the studies reported that the feedback intervention had a positive effect on patient-related outcomes (Table 2 of online ESM), while the remainder reported no change. The 21 studies with positive results centred mostly on improved documentation in areas such as discharge summaries, patient progress notes, prescription writing, and diagnostic capture and coding. Other areas that yielded positive results included patient management skills and patient satisfaction scores. Of the few studies that did not show a positive effect (Table 2 of online ESM), two were related to adherence to practice guidelines with individual studies on adenoma detection rate, immunization rates and obtaining a psychosocial history. There were no commonalities in the group that did not show improvement related to the type of patient outcomes or feedback delivery method.

The most common patient-related outcomes (Table 3 of online ESM) were in the realm of patient management, such as adherence to practice guidelines and immunization rates, and documentation in the areas of medical record-keeping, discharge summaries and diagnostic coding. The patient-related data that were utilized to assess the impact of the feedback interventions most commonly consisted of practice performance parameters such as adherence to practice guidelines and immunization rates. Procedural or exam skills were also frequently assessed and included components such as measurement of liver span, Papanicolaou smear adequacy or adenoma detection rates. Patient-related data were primarily collected through chart review with utilization of a checklist or chart audit tool or by reviewing skills data such as pap smear adequacy. Feedback data were delivered to the participants most frequently through a written format. If the feedback was delivered in-person, the majority involved the authors or another faculty member.

### Participants and setting

The majority of the articles involved one medical discipline at one institution (Table 4 of online ESM). The remaining articles described either medical students at one school, one discipline at multiple institutions or multiple disciplines at one institution. The studies primarily involved internal medicine residents alone and medical students, with the remainder utilizing a variety of other resident specialties. The four studies involving medical students focused on physical exam skills, interviewing techniques and written documentation and all had positive results. There were no significant differences noted between the studies involving students and more advanced trainees. Gastroenterology fellows were utilized in one study while the participants were not clearly identified in the remaining study.

The study settings were primarily academic teaching hospitals with two at Veterans Affairs (VA) teaching hospitals and one at an outpatient multispecialty group practice. Only four of the studies were conducted outside of the United States.

### Methodology of studies

Four of the studies involved a randomized controlled trial study design (Table 5 of online ESM). Most of the investigations were of a quasi-experimental design. The remaining three studies utilized a retrospective category of methodology.

Inclusion and exclusion criteria were specified in approximately half of the studies. A feedback tool was utilized in 14 of the studies. Three of the studies had longer-term follow-up, ranging from 4 months to 5 years.

The sample size of participants ranged from 7 to 96, with roughly half of the studies involving participant sizes less than 30, ten in the 31–49 grouping and two greater than 50. The number of participants was not stated in one study.

## Discussion

Patient outcomes feedback is currently widely used at the practising health professional level as a strategy to improve practice performance both on its own and as a component of multifaceted quality improvement interventions [46]. This type of feedback has been shown to successfully improve physician clinical performance and compliance with quality measures [47], especially when the feedback is provided systematically by a reliable source over time [13]. Less is known about the effect of feedback on patient outcomes to medical learners, an important topic for medical education. In the Kirkpatrick Model [48], designed for evaluating the effectiveness of training across four levels, these outcomes represent the highest level (results).

The findings from this descriptive analysis add to the current medical literature by describing what is known about the association of performance feedback to medical learners with characteristics of patient care. Encouragingly, despite deficiencies in rigor and scope, most studies showed a positive relationship between feedback to medical learners and improvement in patient-related outcomes.

Patient care is broadly defined as “the services rendered by members of the health professions for the benefit of a patient [49]”. Most of the identified studies focused on patient care in the areas of healthcare management and practice performance such as adherence to practice guidelines and immunization rates, improving documentation and procedural and physical examination skills. Although we presume indirect outcomes such as improved documentation and procedural skills translate to improved quality of care, more specific studies would be required to precisely document this assumed benefit. There was very little data on the effectiveness of feedback on more direct patient outcomes. This speaks to the inherent difficulty of designing studies that directly relate feedback to measurable patient care outcomes, involve parameters that are generally agreed to be of prime importance, and provide usable data that aligns with learner competency milestones.

The data obtained show that there are few studies devoted to this topic and most were conducted in the short term with small numbers. Very few of the included studies assessed for bias (unforeseen confounding variables), used assessment tools that had undergone validation studies or involved rigorous study designs. This is perhaps secondary to the belief that medical education research may lend itself more to descriptive and clarification studies than the gold standard of randomized controlled trials (RCTs) [17]. Sullivan, for example, suggests that attention to elements such as the use of a comparison group, adequate numbers, recognizing bias and a thoughtful discussion of limitations may be more important than a focus on a RCT study design in medical education research [50].

The identified studies were primarily conducted at a single university teaching hospital in the US and with a single medical discipline, most commonly internal medicine. It would be necessary to conduct studies in more diverse settings, involving multiple disciplines at multiple institutions to make the findings more generalizable. At the very least, involving a broader spectrum of disciplines in these types of studies would add to the medical feedback literature and inform useful methods for helping learners to meet discipline specific competencies and milestones in patient care.

Research that gives attention to the feedback method and delivery, including the contextual factors surrounding the feedback process [51], may help to facilitate the effectiveness of the patient outcomes information. Aligning outcomes studies with the increasing focus on learner characteristics and perceptions that promote feedback acceptance, as well as the alliance between the teacher and the learner [52], will add to the current medical learner feedback literature. A focus of establishment of standards in measuring patient outcomes will allow providers to collect and share data on outcomes more efficiently, facilitate comparisons that will accelerate improved patient care [53] and guide appropriate patient outcomes goals for learners. Positioning learners as key drivers in their own education and encouraging them to solicit and generate their own patient outcomes feedback may facilitate advancement in this area as well [54].

### Limitations

Overall, the small number of studies included in this analysis and their lack of rigorous study design hamper our ability to draw strong conclusions about the data. It may be difficult to separate the effect of the student impact on patient outcomes from the effect of the team or supervising physician. There is a theoretical risk of publication bias due to the likelihood of studies with positive outcomes being more likely to be submitted and accepted for publication than studies showing no change or a negative outcome. Another limitation is the lack of generalizability due to the preponderance of single discipline, single institution studies.

## Conclusion

Published studies focusing on the association of medical learner feedback with characteristics of patient care are sparse. Most involve a single discipline at a single institution and are of a non-rigorous design. Measurements of improved patient outcomes are restricted to changes in management, procedures and documentation. Well-designed studies that more directly link learner feedback to patient outcomes may help to support the use of feedback in teaching clinical outcomes improvement in alignment with competency-based milestones.

## Caption Electronic Supplementary Material

Table 1 Summary of References on Medical Learner Feedback and Patient Care

Table 2 Patient outcomes (*n* = 27)

Table 3 Patient-related outcomes (*n* = 27)

Table 4 Participants and setting (*n* = 27)

Table 5 Methodology (*n* = 27)

## References

[1] Ramani S, Krackov SK (2012). Twelve tips for giving feedback effectively in the clinical environment. Med Teach.

[2] Archer JC (2010). State of the science in health professional education: effective feedback. Med Educ.

[3] Anderson PA (2012). Giving feedback on clinical skills: are we starving our young?. J Grad Med Educ.

[4] Holmboe ES, Yamakazi K, Edgar L (2015). Reflections on the first 2 years of milestone implementation. J Grad Med Educ.

[5] Lefroy J, Watling C, Teunissen PW, Brand P (2015). Guidelines: the do’s, don’ts and don’t knows of feedback for clinical education. Perspect Med Educ.

[6] Epstein RM (2007). Assessment in medical education. N Engl J Med.

[7] van de Ridder JMM, Stokking KM, McGaghie WC, ten Cate OT (2008). What is feedback in clinical education?. Med Educ.

[8] Ende J (1983). Feedback in clinical medical education. JAMA.

[9] The Institute for Healthcare Improvement (IHI) (2017). The IHI Triple Aim.

[10] Nasca TJ, Philbert I, Brigham T, Flynn TC (2012). The next GME accreditation system – rationale and benefits. N Engl J Med.

[11] Hamstra SJ, Edgar L, Yamazaki K, Holmboe ES (2016). Milestones annual report 2016. Accreditation council for graduate medical education.

[12] Porter ME (2009). A strategy for healthcare reform – toward a value-based system. N Engl J Med.

[13] Veloski J, Boex JR, Grasberger MJ, Evans A, Wolfson DB (2006). Systematic review of the literature on assessment, feedback and physicians’ clinical performance: BEME Guide No. 7. Med Teach.

[14] Bing-You R, Hayes V, Varaklis K (2017). Feedback for learners in medical education: What is known? A scoping review. Acad Med.

[15] Arksey H, O’Malley L (2005). Scoping studies: towards a methodological framework. Int J Social Res Method.

[16] Gough D, Thomas J, Oliver S (2012). Clarifying the differences between review designs and methods. Syst Rev.

[17] Gordon M, Gibbs T (2014). STORIES statement: Publication standards for healthcare education evidence synthesis. BMC Med.

[18] Moher D, Liberati A, Tetzlaff J, Altman DG (2009). Preferred reporting items for systematic reviews and meta-analyses: the PRISMA statement. Ann Intern Med.

[19] Arntfield RT (2015). The utility of remote supervision with feedback as a method to deliver high-volume critical care ultrasound training. J Crit Care.

[20] Axon RN, Penney FT, Kyle TR (2014). A hospital discharge summary quality improvement program featuring individual and team-based feedback and academic detailing. Am J Med Sci.

[21] Barloon TJ, Brown BP, Abu-Yousef MM (1998). Teaching physical examination of the adult liver with use of real-time sonography. Acad Radiol.

[22] Bhatia RS, Milford CE, Picard MH, Weiner RB (2013). An educational intervention reduces the rate of inappropriate echocardiograms on an inpatient service. JACC Cardiovasc Imaging.

[23] Boekeloo BO, Becker DA, Levine DM (1990). Strategies for increasing house staff management of cholesterol with inpatients. Am J Prev Med.

[24] Brody DS (1980). Feedback from patients as a means of teaching nontechnological aspects of medical care. J Med Educ.

[25] Cope DW, Linn LS, Leake B, Barrett PA (1985). Modification of residents’ behavior from patients. Clin Res.

[26] El Saadawi, Tseytlin E, Legowski E (2008). A natural language intelligent tutoring system for training pathologists – implementation and evaluation. Adv Health Sci Educ Theory Pract.

[27] Fairbairn S, Maguire P, Chambers H, Sanson-Fischer R (1983). The teaching of interviewing skills: comparison of experienced and novice trainers. Med Educ.

[28] Goebel LJ (1997). A peer review feedback method of promoting compliance with preventive care guidelines in a resident ambulatory care clinic. Jt Comm J Qual Improv.

[29] Holmboe E, Scranton R, Sumption K, Hawkins R (1998). Effect of medical record audit and feedback on residents’ compliance with preventive healthcare guidelines. Acad Med.

[30] Jin EH, Im JP, Kim JS (2015). The effect of periodic feedback on screening colonoscopy for colorectal polyp and adenoma detection rate in gastroenterology fellows. Gut.

[31] Kim D, Spellberg B (2014). Does real-time feedback to residents with or without attendings improve medical documentation?. Hosp Pract.

[32] Kogan JR, Reynolds EE, Shea JA (2003). Effectiveness of report cards based on chart audits of residents’ adherence to practice guidelines on practice performance: a randomized controlled trial. Teach Learn Med.

[33] Leber M, He C, Akhtar S (2012). A comparison of individualized feedback versus standard didactic lecture to teach interpersonal communication skills to emergency medicine residents: a multicenter randomized controlled trial. Ann Emerg Med.

[34] Maguire P, Fairbairn S, Fletcher C (1986). Consultation skills of young doctors: benefits of feedback training in interviewing as students persist. BMJ.

[35] Mayefsky JH, Foye HR (1993). Use of a chart audit: teaching well child care to pediatric house officers. Med Educ.

[36] Miyakis S, Karamanof G, Liontos M, Mountokalakis T (2006). Factors contributing to inappropriate ordering of tests in an academic department and the effect of an educational feedback strategy. Postgrad Med J.

[37] Niehaus AH, York NL, DaRosa DA, Markwell SJ, Folse R (1995). The effect of feedback on students’ abilities to write daily progress notes. Teach Learn Med.

[38] Opila DA (1997). The impact of feedback to medical housestaff on chart documentation and quality of care in the outpatient setting. J Gen Intern Med.

[39] Quill TE (1985). Outpatient chart review: evidence for preceptor/resident modeling and a mechanism for feedback. Clin Res.

[40] Rust CT, Sisk FA, Kuo AR, Smith J, Miller R, Sullivan KM (1999). Impact of resident feedback on immunization outcomes. Arch Pediatr Adolesc Med.

[41] Shaughnessy AF, D’Amico F (1994). Long-term experience with a program to improve prescription-writing skills. Fam Med.

[42] Simon SS, Soumerai SB (2005). Failure of internet-based audit and feedback to improve quality of care delivered by primary care residents. Int J Qual Health Care.

[43] Sorita A, Steinberg DI, Leitman M, Burger A, Husk G, Sivaprasad L (2014). The assessment of stat laboratory test ordering practice and impact of targeted individual feedback in an urban teaching hospital. J Hosp Med.

[44] Watkins RS, Moran WP (2004). The impact of targeted resident education and feedback on pap smear adequacy rates. J Gen Intern Med.

[45] Wegner A, Campbell L, Shi Y, Fletcher K (2015). Feedback as a tool for promoting high quality documentation among internal medicine residents. J Gen Intern Med.

[46] Ivers N, Jamtvedt G, Flottorp S, Young J (2012). Audit and feedback: effects on professional practice and healthcare outcomes. Cochrane Database Syst Rev.

[47] Loy V, Kwiatt J, Dodda A, Martin E, Dua A, Saeian K (2016). Performance feedback improves compliance with quality measures. Am J Med Qual.

[48] Yardley S, Dornan T (2012). Kirkpatrick’s levels and education ‘evidence’. Med Educ.

[49] http://medical-dictionary.thefreedictionary.com/Patient+care. Retrieved 7 February 2017.

[50] Sullivan GM (2011). Getting off the ‘gold standard’: randomized controlled trials and education research. J Grad Med Educ.

[51] Watling C (2014). Cognition, culture and credibility: deconstructing feedback in medical education. Perspect Med Educ.

[52] Bowen L, Marshall M, Murdoch-Eaton D (2017). Medical student perceptions of feedback and feedback behaviors within the context of the ‘educational alliance.’. Acad Med.

[53] Porter ME, Larsson S, Lee TH (2016). Standardizing patient outcomes measurement. N Engl J Med.

[54] Boud D, Molloy E (2013). Rethinking models of feedback for learning: the challenge of design. Assess Eval High Educ.

