# Supplementary material for: Is feedback to medical learners associated with characteristics of improved patient care?
Source: Perspect Med Educ. 2017 Aug 29;6(5):319–24. doi: 10.1007/s40037-017-0375-8 (PMC5630536; doi:10.1007/s40037-017-0375-8)
Supplement: Supplementary file 1 — Table 1 Summary of References on Medical Learner Feedback and Patient Care [file 40037_2017_375_MOESM1_ESM.docx]

**Table 1. Summary of References on Medical Learner Feedback and Patient Care**

| **Author, Year, Reference** | **Setting & Location** | **Participants** | **Study Design** | **Major Study Findings** |
| --- | --- | --- | --- | --- |
| Arntfield,  2015, (19) | Western University, Ontario, Canada | Internal medicine residents | Retrospective  descriptive | 29 trainees completed 2531 ultrasound exams in 3 ICU’s  23/29 trainees obtained ultrasound reading competency with >80% offline feedback |
| Axon, 2014, (20) | Ralph H Johnson VA Medical Center,  Charleston, SC | Internal medicine residents | Quasi-experimental:  uncontrolled before and after | 3-month pre-intervention assessment and 3-month post-assessment  Improved mean scores on discharge summary quality template scores from 70% to 82% (p=0.05) |
| Barloon, 1998, (21) | University of Iowa Carver College of Medicine, Iowa City, IA | 2^nd^ year medical students | Quasi-experimental:controlled before and after | Students assigned by lottery to intervention group with US or control group  Significant improvement (decrease in mean differences in liver exam measurements from standard) in ultrasound feedback group (p=0.05) |
| Bhatia, 2013, (22) | Harvard Medical School-Mass General Hospital, Boston, MA | Internal medicine residents | Quasi-experimental:  time series | Educational intervention on transthoracic echo-cardiogram ordering  Reduced inappropriate ordering of TEE by 26% (p=0.001) |
| Boekeloo, 1990, (23) | Johns Hopkins University School of Medicine, Baltimore, MD | Internal medicine residents | Experimental: randomized controlled trial | 29 interns randomly assigned to 4 intervention groups (control, reminder checklists, patient-specific feedback, or both)  Greater improvement in inpatient cholesterol management mean rank score in patient-specific feedback groups (p=0.001) |
| Brody, 1980, (24) | Case Western Reserve University School of Medicine, Cleveland, OH | Internal medicine residents | Quasi-experimental:uncontrolled before and after | No improvement in recognition of psychosocial aspects of patient history after minimum of 4 feedback discussions with author  Resident survey showed 76% believed the feedback led to changes in communication with patients |
| Cope, 1985, (25) | David Geffen School of Medicine at UCLA, Los Angeles, CA | Internal medicine residents | Quasi-experimental:controlled before and after | Feedback from patients’ evaluations of art and technical quality of care  Greater improvement in patient satisfaction scores in feedback group vs non-feedback group (p<0.001), though both groups improved |
| El Saadawi, 2008, (26) | University of Pittsburgh School of Medicine, Pittsburgh, PA | Pathology residents | Quasi-experimental:controlled before and after | Feedback from an intelligent tutoring system  4-fold improvement in accuracy of pathology reports in feedback group (p<0.05)  Most learning gain in first period and correlated with number of cases viewed. |
| Fairbairn, 1983, (27) | School of Medicine, University of Man-chester, UK | 4^th^ year medical students on psychiatry rotation | Quasi-experimental: uncontrolled before and after | Random allocation to one of 3 teachers of interviewing skills  Improved interviewing rating skills in all participants (p<0.05) |
| Goebel, 1997, (28) | Marshall University School of Medicine, WV | Internal medicine residents | Quasi-experimental: time series | Peer review feedback program  Improved compliance with 4 of 9 preventive care guidelines during feedback period and all 9 in follow-up period (p<0.05) |
| Holmboe, 1998, (29) | Naval Medical Center Ports-mouth, VA | Internal medicine residents | Retrospective comparative | Chart audit and feedback on preventive health interventions  Improved compliance with audited (as well as one non-audited) preventive care guidelines (p<0.05) |
| Jin, 2015, (30) | Seoul National University Hospital, Korea | Gastro-enterology fellows | Quasi-experimental: time series | Feedback of adenoma detection rates  No difference in overall detection rates for first 150 cases  Significant difference in adenoma detection rate noted after 150 colonoscopies with or without feedback (p<0.05) |
| Kim, 2014, (31) | Harbor-UCLA Medical Center, CA | Internal medicine residents | Quasi-experimental: uncontrolled before and after | Random feedback to residents alone or with attendings on documentation  Improved diagnosis capture and severity of illness scoring (p<0.001)  No difference between resident alone or resident/attending groups |
| Kogan, 2003, (32) | University of Penn-sylvania Health System, PA | Internal medicine residents | Experimental: randomized controlled trial | Report cards based on chart audits for preventive health practices  No significant difference between feedback intervention group and control group |
| Leber, 2012, (33) | St. Luke’s-Roosevelt Hospital Center, Beth Israel Medical Center, Albany Medical Center, NY | Emergency medicine residents | Quasi-experimental: controlled before and after | Individualized feedback on communication skills vs traditional didactic format  Improved patient questionnaire scores on interpersonal skills by 7.6% for intervention group vs 2.4% for control group |
| Maguire, 1986, (34) | School of Medicine, University of Man-chester, Withington Hospital, UK | 4^th^ year medical students on psychiatry rotation | Experimental: randomized controlled trial | Improved interviewing skills persist at 5-year follow-up for video feedback group vs conventional teaching (p<0.001) |
| Mayefsky,1993, (35) | University of Rochester General Hospital, NY | Pediatric residents | Quasi-experimental: controlled before and after | Chart audit feedback vs control group with no feedback  Improvement in utilization of 3 components of well child care in chart audit group (p<0.05) only |
| Miyakis, 2006, (36) | Sotiria General Hospital, Athens School of Medicine, Greece | Unclear: “junior and senior trainees” | Quasi-experimental: uncontrolled before and after | Chart audit with feedback on test-ordering behavior  Decrease in number of avoidable tests ordered after feedback (p=0.002) |
| Niehaus, 1995, (37) | Southern Illinois University School of Medicine, IL | 3^rd^ year medical students on surgery rotation | Quasi-experimental:controlled before and after | Feedback on written progress notes  Improved scores on assessment and plan portion but not subjective data (p<0.05) |
| Opila, 1997, (38) | Health Services Research Group, St. Joseph’s Hospital and Medical Center, AZ | Internal medicine residents | Quasi-experimental: uncontrolled before and after | Chart audit with feedback on documentation and subjective quality of care  Improved scaled chart documentation score from 0.6 to 0.86 (p<0.001) but no improvement in quality of care score |
| Quill, 1985 (39) | University of Rochester School of Medicine, NY | Internal medicine residents | Retrospective descriptive | Random chart selection audit  Resident documentation of health maintenance poor (6% complete, 17% partial, 77% absent from records) and paralleled attendings |
| Rust, 1999 (40) | Emory University School of Medicine, GA | Pediatric residents | Experimental: randomized controlled trial | Retrospective written feedback  No change in immunization rates after intervention (p=0.34) |
| Shaugh-nessy, 1994 (41) | Harrisburg Hospital, Penn State College of Medicine, PA | Family medicine residents | Quasi-experimental:uncontrolled before and after | Feedback on prescription-writing skills  Decrease in prescription-writing errors from 14% to 6% (p=0.0002) over 2-year period |
| Simon, 2005, (42) | Harvard Vanguard Medical Associates, MA | Internal medicine residents | Quasi-experimental: time series | Internet-based audit and feedback  Only 4 of 12 residents accessed their audit data  No change in adherence to practice guidelines for hypertension or diabetes |
| Sorita, 2014, (43) | Beth Israel Deaconess Medical Center, MA | Internal medicine and surgery residents | Quasi-experimental:uncontrolled before and after | Targeted individualized feedback on stat lab ordering  Decreased inappropriate stat lab ordering from 63% to 49% (p=0.004) in feedback group  No change in control group |
| Watkins, 2004, (44) | Wake Forest School of Medicine, NC | Internal medicine residents | Quasi-experimental:controlled before and after | Educational workshop and peer comparison feedback on pap smear adequacy rates  Intervention group twice as likely as control group to obtain adequate pap smear [84 vs 70%] (p=0.02) |
| Wegner, 2015, (45) | Medical College of Wisconsin, WI | Internal medicine residents | Quasi-experimental:uncontrolled before and after | Audit and feedback of quality of inpatient progress notes using a scoring tool (PDQI-9)  Improved quality score of inpatient progress notes (p=0.002) in 4 of 10 attributes |
