# Supplementary material for: Is feedback to medical learners associated with characteristics of improved patient care?
Source: Perspect Med Educ. 2017 Aug 29;6(5):319–24. doi: 10.1007/s40037-017-0375-8 (PMC5630536; doi:10.1007/s40037-017-0375-8)
Supplement: Supplementary file 2 — Table 2 Patient outcomes (n = 27) [file 40037_2017_375_MOESM2_ESM.docx]

| **Outcomes** | **Sample n (%)** |
| --- | --- |
|  |  |
| Positive | 22 (82%) |
| Documentation^20,26,31,35,37,38,40,45^ | 8 (30%) |
| Patient management skills |  |
| - Preventive care measures^23,28,29,39^ | 4 (15%) |
| - Communication skills^27,33,34^ | 3 (11%) |
| - Appropriate test ordering^22,336,43^ | 3 (11%) |
| - Physical exam skills^19,21,44^ | 3 (11%) |
| Patient satisfaction^25^ | 1 (4%) |
|  |  |
| No effect | 5 (18%) |
| Adherence to practice guidelines^32,42^ | 2 (7%) |
| Adenoma detection rates^30^ | 1 (4%) |
| Immunization rates^40^ | 1 (4%) |
| Communication skills^24^ | 1 (4%) |
|  |  |

**Table 2.** Patient outcomes (n=27)
