# Supplementary material for: Is feedback to medical learners associated with characteristics of improved patient care?
Source: Perspect Med Educ. 2017 Aug 29;6(5):319–24. doi: 10.1007/s40037-017-0375-8 (PMC5630536; doi:10.1007/s40037-017-0375-8)
Supplement: Supplementary file 3 — Table 3 Patient-related outcomes (n = 27) [file 40037_2017_375_MOESM3_ESM.docx]

**Table 3.** Patient-related outcomes (n=27)

| **Parameter** | **Sample n (%)** |
| --- | --- |
| *Outcome objectives* |  |
| - Improved documentation^20,31,35,37,38,41,45^ | 7 (26%) |
| - Adherence to practice guidelines^23,28,29,32,42^ | 5 (19%) |
| - Improved communication skills^24,27,33,34^ | 4 (15%) |
| - Improved procedural skills^19,26,44^ | 3 (11%) |
| - Appropriate laboratory test ordering^22,36,43^ | 3 (11%) |
| - Improved physical exam skills^21,30^ | 2 (7%) |
| - Improved prescription-writing accuracy^41^ | 1 (4%) |
| - Improved patient satisfaction^25^ | 1 (4%) |
| - Increased Immunization rates^40^ | 1 (4%) |
| *Patient-related data assessed* |  |
| - Practice performance parameters^23,24,28,29,32,35,38-40,42^ | 10 (37%) |
| - Procedural or exam skills^19,21,26,30,41,44^ | 6 (22%) |
| - Written documentation^20,31,37,45^ | 4 (15% |
| - Communication skills^27,33,34^ | 3 (11%) |
| - Laboratory tests ordered^22,36,43^ | 3 (11%) |
| - Patient satisfaction scored^25^ | 1 (4%) |
| *Data collection method* |  |
| - Chart review^20,23,24,28,29,31,32,35-40,43,45^ | 15 (56%) |
| - Direct observation^19,21,30,44^ | 4 (15%) |
| - Videotape reviews^27,43^ | 2 (15%) |
| - Patient surveys^25,33^ | 2 (15%) |
| - Written documents^26,42^ | 2 (15%) |
| - Diagnostic test ordering assessment^22^ | 1 (11%) |
| - Frequency of accessing internet site^42^ | 1 (11%) |
| *Feedback delivery method* |  |
| - Written format^19,23,28,33,35,37,40,41^ | 8 (30%) |
| - Individual one-on-one^31,32,38,43^ | 4 (15%) |
| - Electronically^22,26,30,42^ | 4 (15%) |
| - During clinic session^24,25,39^ | 3 (11%) |
| - Group training session^20,21,27^ | 3 (11%) |
| - Discussion utilizing videotaped^34^ | 1 (4%) |
| - Method unclear^29,36,44,45^ | 4 (15%) |
| *Who delivered feedback* |  |
| - Authors^21,22,24,31,42^ | 5 (19%) |
| - Other faculty^20,32,37,38^ | 4 (15%) |
| - Additional professional personnel^23,27,41^ | 3 (11%) |
| - Peers^28^ | 1 (4%) |
| - Residency director^43^ | 1 (4%) |
| - Interface tutoring system^26^ | 1 (4%) |
| - Not discussed in person^19,29,33,39,40,44^ | 6 (22%) |
| - Unclear^25,30,34-36,45^ | 6 (22%) |
