# Supplementary material for: Is feedback to medical learners associated with characteristics of improved patient care?
Source: Perspect Med Educ. 2017 Aug 29;6(5):319–24. doi: 10.1007/s40037-017-0375-8 (PMC5630536; doi:10.1007/s40037-017-0375-8)
Supplement: Supplementary file 4 — Table 4 Participants and setting (n = 27) [file 40037_2017_375_MOESM4_ESM.docx]

| **Parameter** | **Sample n (%)** |
| --- | --- |
| *# Disciplines and institutions* |  |
| - One discipline at one institution^20,22-26,28-31,36,38-42,44,45^ | 18 (67%) |
| - Medical students at one school^21,27,34,37^ | 4 (15%) |
| - One discipline at multiple institutions^32,33,35^ | 3 (11%) |
| - Multiple disciplines at one instituion^19,43^ | 2 (7%) |
| *Type of learner* |  |
| - Internal medicine residents^19,20,22-25,28,29,31,32,38,39,42,44,45^ | 15 (56%) |
| - Medical students^21,27,34,37^ | 4 (15%) |
| - Pediatric residents^35,40^ | 2 (7%) |
| - Family Medicine residents^41^ | 1 (4%) |
| - Pathology residents^26^ | 1 (4%) |
| - Emergency medicine residents^33^ | 1 (4%) |
| - Gastroenterology fellows^30^ | 1 (4%) |
| - Internal medicine and surgery residents^43^ | 1 (4%) |
| - Unclear^36^ | 1 (4%) |
| *Setting* |  |
| - Academic medical center^19,21-28,30-33,35-41,43-45^ | 24 (89%) |
| - Veterans Affairs teaching hospital^20,29^ | 2 (7%) |
| - Outpatient multi-specialty practice^42^ | 1 (4%) |
| *Location* |  |
| - United States^20-26,28,29,31-33,35,37-45^ | 22 (81%) |
| - United Kingdom^27,34^ | 2 (7%) |
| - Canada^19^ | 1 (4%) |
| - Greece^36^ | 1 (4%) |
| - Korea^30^ | 1 (4%) |

**Table 4.** Participants and setting (n=27)
