# Supplementary material for: Is feedback to medical learners associated with characteristics of improved patient care?
Source: Perspect Med Educ. 2017 Aug 29;6(5):319–24. doi: 10.1007/s40037-017-0375-8 (PMC5630536; doi:10.1007/s40037-017-0375-8)
Supplement: Supplementary file 5 — Table 5 Methodology (n = 27) [file 40037_2017_375_MOESM5_ESM.docx]

| **Parameters** | **Sample n (%)** |
| --- | --- |
|  |  |
| *Design* |  |
| Randomized controlled trial^23,32,24,40^ | 4 (15%) |
| Quasi-experimental |  |
| - Uncontrolled before and after^20,24,27,31,36,38,41,43,45^ | 9 (45%) |
| - Controlled before and after^21,25,26,33,35,37,44^ | 7 (35%) |
| - Time series^22,28,30,42^ | 4 (20%) |
| Retrospective^19,29,39^ | 3 (11%) |
|  |  |
| Exclusion criteria specified^24,25,41,34-37,40-42,43,44^ | 13 (48%) |
|  |  |
| Use of feedback tool^19,20,23,25,27,28,31,33,34,37,38,40-42^ | 14 (52%) |
|  |  |
| Long-term follow-up^22,34,35^ | 3 (11%) |
|  |  |
| *Sample size* |  |
| - Less than 30^19,23,25,26,29-31,35-38,41-43^ | 14 (52%) |
| - 31-49^21,22,24,27,28,31,33,35,39,40^ | 10 (37%) |
| - 50 or more^20,45^ | 2 (7%) |
| - not specified^34^ | 1 (4%) |
|  |  |
| Power analysis conducted^31^ | 1 (4%) |
|  |  |
| Mention of bias^26,27^ | 2 (7%) |

**Table 5.** Methodology (n=27)
